# Detection and Genetic Analysis of Noroviruses and Sapoviruses in Sea Snail

**DOI:** 10.1007/s12560-015-9205-5

**Published:** 2015-06-23

**Authors:** Hiroki Ozawa, Makoto Kumazaki, Satoshi Ueki, Masahiro Morita, Shuzo Usuku

**Affiliations:** Department of Testing and Research, Yokohama City Institute of Health, Tomiokahigashi 2-7-1, Kanazawa-ku, Yokohama, Kanagawa 236-0051 Japan

**Keywords:** Norovirus, Sapovirus, Sea snail, Phylogenetic analysis

## Abstract

An outbreak of acute gastroenteritis occurred at a restaurant in Yokohama in December 2011. Because many of the customers had consumed raw sea snail, sea snail was suspected to be the source of this outbreak. To determine whether sea snail contains *Norovirus* (NoV) or *Sapovirus* (SaV), we analyzed 27 sea snail samples collected over 5 months (May, June, August, October, and December 2012) and 59.3 % were positive for NoV and/or SaV. The levels of NoV ranged from 1.5 × 10^3^ to 1.5 × 10^5^ copies/g tissue, and those of SaV from 1.5 × 10^2^ to 1.3 × 10^3^ copies/g tissue. The highest levels were observed in sea snails collected in December. A phylogenetic analysis of the NoVs showed that the viral strains were NoV genotypes GI.4, GI.6, GII.4, GII.12, GII.13, and GII.14, and the SaV strains were genotypes GI.2 and GI.3. The NoV GII.4 Sydney 2012 variants were only detected in December. This variant was a major source of gastroenteritis in Japan in the winter of 2012/2013. In contrast, the NoV GII.4 strains detected in May and June 2012 were not the Sydney 2012 variant. This study demonstrates that sea snail contains multiple genogroups and genotypes of NoV and SaV strains. We conclude that the sea snail presents a risk of gastroenteritis when consumed raw.

## Introduction

Acute gastroenteritis caused by *Norovirus* (NoV) or *Sapovirus* (SaV) is a common infectious disease. These viruses are classified as caliciviruses, and have single-stranded positive-sense 7.5-kb RNA genomes (Berke et al. 1997; Wilhelmi et al. 2003; Patel et al. 2009; Morillo and Timenetsky 2011). Both NoV and SaV are classified into I–VI genogroups. The NoVs responsible for human gastroenteritis occur in genogroup I (GI), GII, and GIV (Zheng et al. 2006; Wang et al. 2007; Miura et al. 2013). Of the many genotypes that exist, genotype GII.4 causes most human outbreaks (Takanashi et al. 2011). The characteristic incubation period for NoV and SaV infections is 1–2 days, and the major clinical symptoms are diarrhea, vomiting, nausea, and abdominal cramps (Patel et al. 2009). Oysters are a known vehicle of NoV transmission, and their raw consumption causes gastroenteritis outbreaks attributable to NoV infection (Baker et al. 2011).

Bivalves, such as oysters, filter large amounts of water, thus any human pathogen in sewage can bioaccumulate in their digestive tissues (Le Guyader et al. 2010; Schaeffer et al. 2013). Because contamination of shellfish is related to sewage (Wang and Deng 2012; Prato et al. 2013), consumption of raw bivalves is a potential cause of viral gastroenteritis (Prato et al. 2004).

In 2011, an outbreak of acute gastroenteritis at a restaurant was reported in the Public Health Center, Yokohama. Because plural NoVs and SaVs were detected from the stools of the patients, bivalves were suspected as the cause of the food-borne gastroenteritis. However, the restaurant had supplied raw sea snails instead of bivalves to prevent norovirus infection. In Japan, sea snail is sold at fish markets and is eaten raw or boiled, although it is rarely eaten raw in Japan or other countries. As far as we know, the detection of NoV or SaV in sea snail has not been reported. Bivalves such as the oyster are known to bioaccumulate pathogens that are major etiological agents of gastroenteritis. However, compared with bivalves, sea snail has been considered to present little risk of NoV infection. The aim of this study was to determine whether NoV or SaV is detectable in sea snails. We also investigated the concentrations of NoV and SaV contamination in the sea snail and constructed a phylogenetic tree to characterize these viruses.

## Materials and Methods

### Sea Snail Samples

**Fig. 1:**
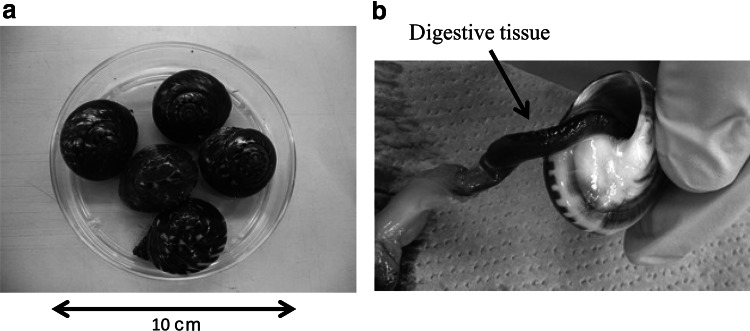
**a** Shells of the sea snail *Umbonium giganteum*. **b** Digestive tissue of the sea snail

An outbreak of acute gastroenteritis occurred at a restaurant in Yokohama in December 2011. An investigation revealed that 71 (37.0 %) of 192 customers displayed symptoms of gastroenteritis. NoVs were detected in 24 (70.6 %) of the 34 stool specimens tested. Many of these customers had consumed raw sea snail *Umbonium giganteum* (Fig. 1a), which is a marine filter-feeding gastropod mollusk of the family Trochidae. Therefore, this sea snail was suspected of being the source of the outbreak. After the outbreak, 27 sea snail samples were collected in May, June, August, October, and December 2012 from Kanagawa Prefecture, in the coastal region from which the sea snails had been supplied to the restaurant. Five sea snails were obtained in May, June, August, and October, and seven in December. Although the weight of the sea snails differed depending on individuals or collection periods, each sea snail was approximately 10–20 g. The samples were transported to the laboratory about 1 day after their collection, where their digestive tissues (Fig. 1b), which were separated from other tissues, were cut into two portions. One of the two portions was weighed (5.0 × 10^−2^–6.0 × 10^−1^ g: average 2.5 × 10^−1^ g) and then homogenized separately with a Multi-beads Shocker^®^ (Yasui Kikai, Osaka, Japan).

### RNA Extraction and cDNA Synthesis

Viral RNA was extracted from the homogenized sea snail samples with a Qiagen RNeasy Mini Kit (Qiagen, Hilden, Germany) in a final volume of 30 μl, according to the manufacturer’s instructions. The cDNA (50 μl) was synthesized with SuperScript III Reverse Transcriptase (Invitrogen, Carlsbad, CA, USA) and random hexamer primers (Takara Bio Inc., Shiga, Japan). Fifty μl of cDNA was synthesized from 5 μl of the extracted viral RNA in October, and from 10 μl of RNA in May, June, August, and December. The reverse transcription (RT) reaction was incubated at 42 °C for 60 min and then at 95 °C for 5 min to inactivate the enzyme. The amount of cDNA used for real-time PCR was 2.5 μl of the sample or control.

### Real-Time PCR to Quantify NoV and SaV

Real-time PCR amplification of the NoV and SaV genomes was performed with a StepOne™ Real-Time PCR System (Applied Biosystems, Foster City, CA, USA), using a QuantiTect Probe PCR Kit (Qiagen). The optimized primers used for NoV GI were NV192 and NV193, and the probe was TM9 (Hoehne and Schreier 2006); for NoV GII, the primers were JJV2F and COG2R (Jothikumar et al. 2005), and the probe was GII (Hymas et al. 2007). The concentrations of the primers and probes were 900 and 300 nM, respectively. The primers and probes used for SaV were the same as described previously (Oka et al. 2006), and the concentrations of the primers and probes were 400 nM and 200 nM, respectively. The reaction mixture was subjected to the following amplification conditions: for NoV, 2 min at 50 °C, 15 min at 95 °C, 50 cycles of 15 s at 94 °C, and 60 s at 60 °C; for SaV, the annealing temperature was at 62 °C instead of 60 °C.

The samples were analyzed with real-time PCR using standard plasmid (pcDNA3.1/V5-His TOPO; Invitrogen) as the positive control to create the standard curve, and nuclease-free water as the negative control. Each viral genome copy number was determined by comparison with tenfold serial dilutions of a standard curve constructed with a standard plasmid. The average quantity of NoV or SaV in two wells was used to determine the number of viral genome copies/g tissue. Only those samples that displayed amplification in two wells were analyzed quantitatively, and the limit of quantification was 100 copies/g tissue (Manso and Romalde 2013).

In separate experiments, the presence of PCR inhibitors was evaluated by adding internal controls. Each volume of sample RNA extract (5–10 μl) was reverse transcribed with 2.5 μl containing NoV GI RNA as internal controls (a rather high concentration; 2.9 × 10^6^ copies/2.5 μl) (Costafreda et al. 2006; Le Guyader et al. 2008). Nuclease-free water was used in the RT-reaction as the negative control. Efficiency of PCR was performed with the same conditions of real-time PCR for NoV GI.

### PCR for NoV and SaV

Semi-nested PCR for NoV GI was performed using primers COG1F and G1-SKR for the first PCR, and G1-SKF and G1-SKR for the second PCR. For NoV GII, primers COG2F and G2-SKR were used for the first PCR, and G2-SKF and G2-SKR for the second PCR. These primers were used to amplify parts of the polymerase gene and the capsid region, respectively (Kojima et al. 2002; Kageyama et al. 2003). Nested PCR for SaV was performed using a mixture of forward primers (SV-F13 and SV-F14) and reverse primers (SV-R13 and SV-R14) for the first PCR, and forward primer SV-F22 and reverse primer SV-R2 for the second PCR (Okada et al. 2006).

To reduce the risk of contamination, we used separate working areas to prepare the PCR mixes, to add the templates, and to perform the PCR reactions. We also included a no-template control in all PCR reaction series to confirm the absence of contamination.

### Sequencing and Phylogenetic Analyses

The PCR products were purified for sequence analysis with a Montage DNA Gel Extraction Kit (Merck Millipore, Darmstadt, Germany), according to the manufacturer’s instructions. The nucleotide sequences of the purified products (QIAquick PCR Purification Kit, Qiagen) were determined with a BigDye Terminator v1.1 Cycle Sequencing Kit (Applied Biosystems), Centri-Sep™ Spin Columns (Princeton Separations, Adelphia, NJ, USA), and a Genetic Analyzer 3130 (Applied Biosystems). Multisequence alignments were generated with ClustalW (Thompson et al. 1994), and a neighbor-joining phylogenetic tree was constructed with MEGA 5, with 1000 bootstrap replicates.

NoV genotyping was performed as previously described (Kroneman et al. 2013), and genotypes were assigned using a publicly accessible typing tool (http://www.rivm.nl/mpf/norovirus/typingtool) (Kroneman et al. 2011).

The GenBank accession numbers for the reference strains are as follows: AB042808 (Chiba 407), AF093797 (BS5), X76716 (Bristol), AJ277618 (Wortley), AY113106 (Fayetteville), AY130761 (M7), U65427 (Sapporo), U73124 (Parkville), AF194182 (Stockholm/318), AJ412800 (Chiba/000496F), and DQ366345 (Ehime643).

### Nucleotide Sequence Accession Numbers

The nucleotide sequences determined in this study were deposited in the GenBank database under accession numbers AB934018–AB934035. The reference strains used in this study are described on each phylogenetic tree.

## Results

### Virus Detection

A total of 27 sea snail samples were collected in Kanagawa Prefecture in May–December 2012. A summary of the detection and quantification of NoV and SaV with real-time PCR and conventional PCR is shown in Table 1. Sixteen samples (59.3 %) contained at least one of NoV or SaV. Mixed contamination was present in 11 samples (40.7 %). NoV and SaV were detected in 15 (55.6 %) and 12 (44.4 %) samples, respectively (Table 1). NoV was detected in sea snails in May, June, October, and December. SaV was detected in sea snails in May, October, and December. The proportion of sea snails carrying NoV or SaV was highest in December.

**Table 1:** Estimated concentrations of noro- and sapovirus, and detected genotypes in sea snail samples

Month	Sample name	NoV GI			NoV GII			SaV			Efficiency rate (%)
		Real-time PCR^a^ (Copies/g)	Conventional PCR	Genotype	Real-time PCR^a^(Copies/g)	Conventional PCR	Genotype	Real-time PCR^a^(Copies/g)	Conventional PCR	Genotype	
May-2012	nagarami A	4.0 × 10^2^	−	−	7.9 × 10^3^	−	−	+	+	GI.3	42.7
	nagarami B	−	−	−	1.5 × 10^3^	−	−	−	+	GI.2	38.7
	nagarami C	−	−	−	−	−	−	+	+	GI.3	58.9
	nagarami D	−	+	GI.4	4.0 × 10^3^	+	GII.14	1.5 × 10^2^	−	−	100
	nagarami E	−	−	−	3.3 × 10^3^	+	GII.4	+	−	−	100
Jun-2012	nagarami F	−	−	−	−	+	GII.4	+	−	−	100
	nagarami G	−	−	−	−	−	−	−	−	−	76.7
	nagarami H	−	−	−	−	−	−	−	−	−	51.5
	nagarami I	−	−	−	−	−	−	−	−	−	72.8
	nagarami J	−	−	−	−	−	−	−	−	−	74.9
Aug-2012	nagarami K	−	−	−	−	−	−	−	−	−	56.1
	nagarami L	−	−	−	−	−	−	−	−	−	39.1
	nagarami M	−	−	−	−	−	−	−	−	−	61.7
	nagarami N	−	−	−	−	−	−	−	−	−	24.9
	nagarami O	−	−	−	−	−	−	−	−	−	38.8
Oct-2012	nagarami P	+	−	−	−	−	−	−	−	−	100
	nagarami Q	+	−	−	−	−	−	−	−	−	78.7
	nagarami R	−	−	−	−	−	−	−	−	−	85.3
	nagarami S	−	−	−	−	−	−	−	−	−	63.0
	nagarami T	−	−	−	4.7 × 10^3^	−	−	5.8 × 10^2^	−	−	75.7
Dec-2012	nagarami U	4.5 × 10^3^	+	GI.4	6.9 × 10^4^	+	GII.4	2.8 × 10^2^	−	−	100
	nagarami V	2.7 × 10^2^	−	−	2.2 × 10^4^	+	GII.4	+	−	−	100
	nagarami W	−	+	GI.6	3.2 × 10^4^	+	GII.13	−	+	GI.2	53.0
	nagarami X	+	−	−	−	+	GII.4	−	−	−	50.3
	nagarami Y	1.7 × 10^3^	−	−	5.6 × 10^4^	+	GII.12	−	−	−	91.5
	nagarami Z	4.7 × 10^3^	+	GI.6	7.6 × 10^4^	+	GII.4	3.7 × 10^2^	−	−	39.1
	nagarami AA	2.8 × 10^3^	−	–	1.4 × 10^5^	+	GII.4	1.3 × 10^3^	−	−	49.7

^a^In real-time PCR, Numbers represent copy numbers that displayed amplification in two wells and more than the limit of quantification. + indicated less than the limit of quantification

### Virus Quantification

Six, 11, and five samples of NoV GI, NoV GII, and SaV, respectively, were quantifiable, and were therefore available for quantification. The concentrations of total NoV (GI plus GII) in the sea snails ranged from 1.5 × 10^3^ to 1.5 × 10^5^ copies/g tissue during the period of investigation. The highest quantities of total NoV and SaV were recorded in December, and no NoV was detected in August. Thus, the NoV concentrations in sea snails demonstrated a strong seasonal trend. The quantities of NoV GI ranged from 2.7 × 10^2^ to 4.7 × 10^3^ copies/g tissue; GII ranged from 1.5 × 10^3^ to 1.4 × 10^5^ copies/g tissue; and SaV ranged from 1.5 × 10^2^ to 1.3 × 10^3^ copies/g tissue (Table 1).

Efficiency of PCR was more than 24.9 % in all the samples and was more than 38.7 % in the quantitative samples (Table 1). An RT-negative control and a no-template control produced negative results. The amplification of PCR indicated no conspicuous inhibition.

### Phylogenetic Analysis of Sea Snail Samples

**Fig. 2:**
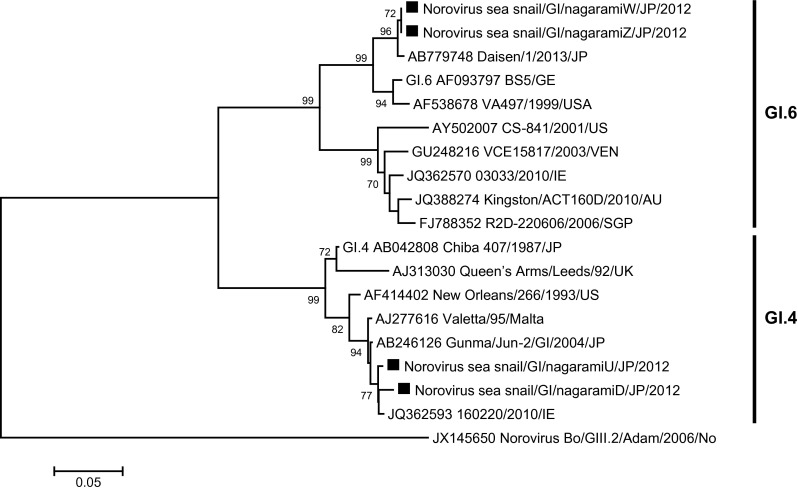
Phylogenetic analysis of Norovirus GI, capsid N/S region. Phylogenetic tree of Norovirus GI strains and reference strains based on the partial capsid sequence, constructed with the neighbor-joining method. The numbers on the branches show only the bootstrap values above 70 %. *Filled square* indicates Norovirus GI strains isolated in this study

**Fig. 3:**
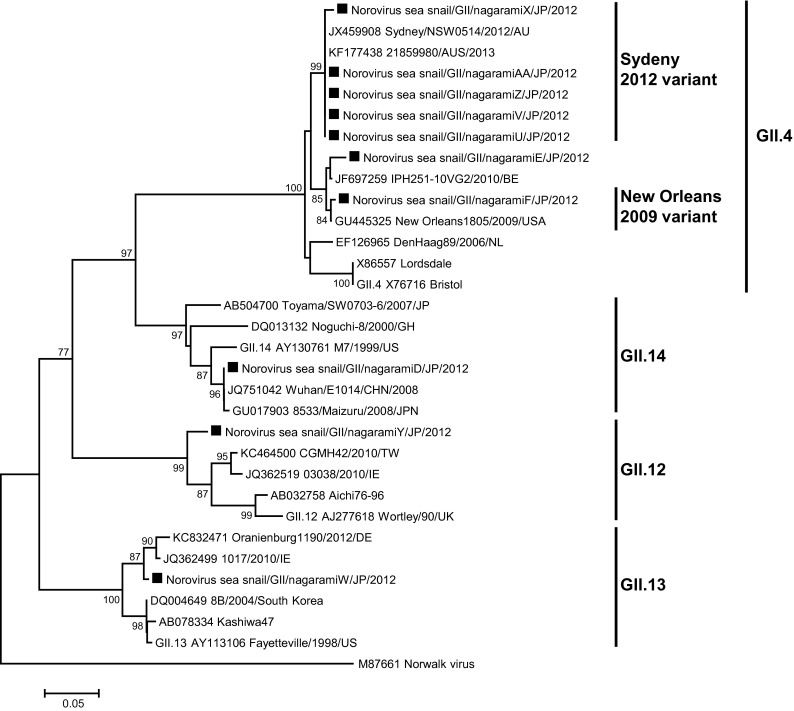
Phylogenetic analysis of Norovirus GII, capsid N/S region. Phylogenetic tree of the Norovirus GII strains and reference strains based on the partial capsid sequence, constructed with the neighbor-joining method. The numbers on the branches show only the bootstrap values above 70 %. *Filled square* indicates Norovirus GII strains isolated in this study

**Fig. 4:**
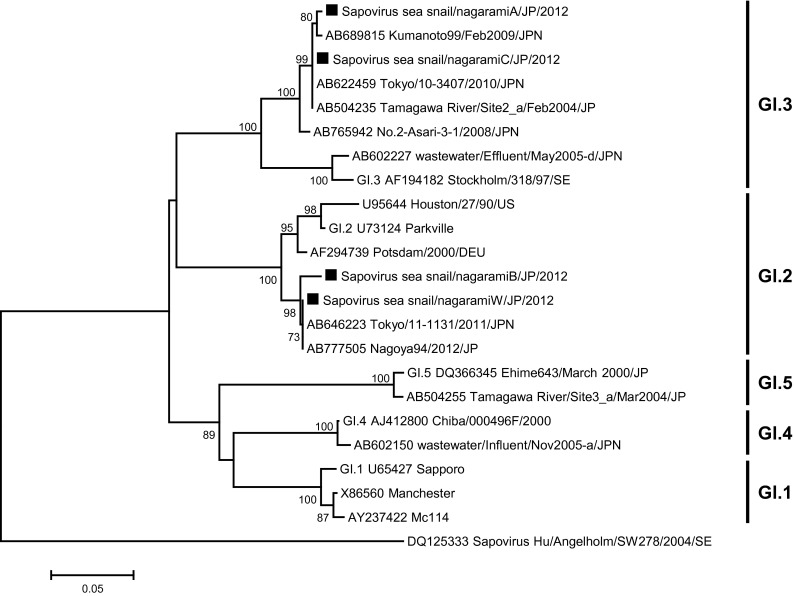
Phylogenetic analysis of Sapovirus, capsid region. Phylogenetic tree of Sapovirus strains and reference strains based on the partial capsid sequence, constructed with the neighbor-joining method. The numbers on the branches show only the bootstrap values above 70 %. *Filled square* indicates Sapovirus strains isolated in this study

Thirteen sea snail samples were NoV- or SaV-positive, as determined with nested PCR, and were confirmed with a sequencing analysis. Phylogenetic analyses of NoV and SaV showed that several viral strains were present during each month. In detail, these genetic types were NoV GI.4 and GI.6 (Fig. 2), NoV GII.4, GII.12, GII.13, and GII.14 (Fig. 3), and SaV GI.2 and GI.3 (Fig. 4). Of the two NoV GI.4 strains, one was collected in May and the other in December. The nucleotide sequence identities of the two NoV GI.4 strains were 98.6 %. The two NoV GI strains detected in December 2012 had 100 % identical sequence and were classified as NoV GI.6. The five NoV GII.4 strains detected in December 2012 were classified as the NoV GII.4 Sydney 2012 variant and showed 99.3–100 % similarity to the NoV GII.4 Sydney 2012 variant reference sequence (JX459908). The other NoV GII.4 strain detected in June was closely related to the NoV GII.4 New Orleans 2009 variant reference sequence (GU445325). The NoV GII.4 Sydney 2012 variants were only detected in December. The NoV GII.4 strain detected in May was not a New Orleans 2009 or Sydney 2012 variant.

The nucleotide sequence identity of the two SaV GI.2 strains was 98.8 %, and that of the two SaV GI.3 strains detected in May was 99.3 %. One of the two SaV GI.2 strains was collected in May and the other in December.

## Discussion

It is well known that bivalves, including oysters, cause acute gastroenteritis. When an oyster grows in contaminated water, it can bioaccumulate multiple pathogens in its digestive tissues, and several viruses have been detected in oyster tissues. Therefore, eating raw shellfish, such as oysters, entails a risk of acute gastroenteritis (Baker et al. 2011). Oyster-associated outbreaks can involve different genetic types of NoV in the same individual (Sugieda et al. 1996), and multiple NoV strains have been detected in oysters (Le Guyader et al. 2008). Another six kinds of bivalves (Manila clams, clams, mussels, razor clams, blood clams, and scallops) can be contaminated with NoVs or SaVs (Iizuka et al. 2010; Xia Ming et al. 2013), and multiple gastroenteric viruses or viral strains have been detected in fecally contaminated shellfish (Gallimore et al. 2005). When an outbreak occurred in Yokohama in 2011, several NoV strains were detected. Although the patients had not eaten bivalves, many of them had eaten raw sea snail. The raw sea snail was pickled in Shochu (Japanese spirit distilled from rice) and eaten, but because NoV is resistant to ethanol, the viruses in the sea snail digestive tissues remained infectious.

The aim of this study was to clarify whether sea snails carry NoV and SaV in their digestive tissues. The sea snail samples examined in this study were collected in May, June, August, October, and December 2012. We could not obtain samples in the other months because sea snail is not harvested from January to April.

Digestive tissue can be a PCR inhibitor and cause false negatives. The presence of PCR inhibitors was evaluated in this study, but evaluation of nucleic extraction efficiencies by adding another external virus as a control was not performed. There is a possibility of underestimating the quantitative results by loss of nucleic extraction or other inhibitors. We used separate working areas and negative controls to avoid the risk of false positives. Our report showed the estimated minimum values of viral contamination in sea snails.

A previous study reported that the 50 % infectious dose of NoV GII was 10^3^ genome copies according to the dose–response relationship in the secretor phenotype population (Teunis et al. 2008). We did not investigate whether the detected viruses were infectious. The NoV GII concentration was higher than the NoV GI concentration, and exceeded 10^3^ copies/g tissue during the investigation period. Therefore, our data show that the NoV GII detected in the sea snail exceeded the infectious dose of NoV for humans. The seasonal distribution indicated that NoV contamination in sea snails is high in December.

A phylogenetic analysis demonstrated that several strains of these viruses were present. The most frequently identified genotype in the sea snail was NoV GII.4. Interestingly, the five NoV GII.4 strains detected in December were the Sydney 2012 variant, which was a major source of the gastroenteritis outbreaks in Japan in the winter of 2012/2013 (Thongprachum et al. 2014). The strains in the other NoV GII.4-positive samples, which were collected in May and June, were not closely related to the Sydney 2012 variant. Mixed contaminations were observed in 11 sea snail samples in this study. These results suggest that this sea snail can bioaccumulate and retain viral pathogens.

In conclusion, we detected both NoV and SaV in sea snail samples. Bivalves are recognized as typical vehicles of food-borne NoV. We conclude that the sea snail presents a risk of gastroenteritis and is a newly recognized vehicle of food-borne NoV and SaV.
